# Understanding traditional and modern eating: the TEP10 framework

**DOI:** 10.1186/s12889-019-7844-4

**Published:** 2019-12-02

**Authors:** Gudrun Sproesser, Matthew B. Ruby, Naomi Arbit, Charity S. Akotia, Marle dos Santos Alvarenga, Rachana Bhangaokar, Isato Furumitsu, Xiaomeng Hu, Sumio Imada, Gülbanu Kaptan, Martha Kaufer-Horwitz, Usha Menon, Claude Fischler, Paul Rozin, Harald T. Schupp, Britta Renner

**Affiliations:** 1 Department of Psychology, University of Konstanz, Konstanz, Germany; 2 Department of Psychology and Counselling, La Trobe University, Albury-Wodonga, Australia; 3 BetterUp, Inc., San Francisco, CA USA; 4 Department of Psychology, School of Social Sciences, University of Ghana, Legon, Accra, Ghana; 5 Department of Nutrition, School of Public Health, University of Sao Paulo, Sao Paulo, Brazil; 6 Department of Human Development & Family Studies, Maharaja Sayajirao University of Baroda, Vadodara, India; 7 Faculty of Health Sciences, Hiroshima-Shudo University, Hiroshima, Japan; 8 Department of Psychology, Tsinghua University, Beijing, China; 9 Centre for Decision Research, Leeds University Business School, University of Leeds, Leeds, LS2 9JT UK; 10 Obesity and Eating Disorders Clinic, Department of Endocrinology and Metabolism, Instituto Nacional de Ciencias Médicas y Nutrición Salvador Zubirán, Mexico City, Mexico; 11 Department of Anthropology, Drexel University, Philadelphia, USA; 12 IIAC, Centre National de la Recherche Scientifique, Paris, France; 13 Department of Psychology, University of Pennsylvania, Philadelphia, USA

**Keywords:** Traditional eating, Modern eating, Conceptual framework, Dietary change, Western diet

## Abstract

Across the world, there has been a movement from traditional to modern eating, including a movement of traditional eating patterns from their origin culture to new cultures, and the emergence of new foods and eating behaviors. This trend toward modern eating is of particular significance because traditional eating has been related to positive health outcomes and sustainability. Yet, there is no consensus on what constitutes traditional and modern eating. The present study provides a comprehensive compilation of the various facets that seem to make up traditional and modern eating. Specifically, 106 facets were mentioned in the previous literature and expert discussions, combining international and interdisciplinary perspectives. The present study provides a framework (the TEP10 framework) systematizing these 106 facets into two major dimensions, what and how people eat, and 12 subdimensions. Hence, focusing only on single facets of traditional and modern eating is an oversimplification of this complex phenomenon. Instead, the multidimensionality and interplay between different facets should be considered to gain a comprehensive understanding of the trends, consequences, and underlying factors of traditional and modern eating.

## Background

We are currently in the midst of a major change in what people eat and in the way they eat [1–4]. Some of these changes have been described as a nutrition transition, which refers to a shift from diets high in complex carbohydrates and fiber towards more varied diets with a higher proportion of fats, saturated fats, and sugar [3, 5–9]. The changes partially result from the globalization and modernization of food and eating, for example, access to new technologies, modern supermarkets, and food marketing [3, 10, 11]. Also, urbanization has separated a large part of the world’s population from the direct production of foods, which has produced changes in eating behavior [12]. Furthermore, these changes have been accompanied by a general increase in wealth and food supply [13] as well as by a decrease in food insecurity [14]. Food safety has improved [15], costs for many foods have decreased [16], and a much wider variety of foods is available to people in almost all parts of the Earth [5]. One result of all of this has been an increase in life expectancy. In the USA, life expectancy increased from 47 years in 1900 to 78 years in 2007, for example [17]. Another advantage of the globalization and modernization of food and eating is that many of the distinctive, nutritious and delicious foods developed by different cuisines, at different localities in the world are now widely available. In a survey of people in 17 countries spanning a wide range of developmental status, 500–2000 individuals per country were asked ‘What is your favorite food?’ [18]. We inspected the five most frequently named foods within these 17 countries and categorized these 85 foods into traditional within the respective country vs. imported from other countries. The results showed that 24 of these foods can be considered traditional in the respective country (e.g., fufu in Ghana, feijoada in Brazil), 29 can be considered foods that have been imported from other parts of the world to the respective country (e.g., pizza and pasta in the Netherlands), and the remaining 32 could not be classified in these two categories (e.g., vegetables in Germany).

At the same time, however, increasing wealth has promoted eating away from home and obesity has increased. The latter will probably affect more people than food insecurity [19] at some point in the next few decades. Also, obesity already co-exists together with food insecurity [20, 21]. As a result of the forces described, there has been a shift from acute, infectious diseases to chronic, degenerative diseases (the epidemiological revolution, [22, 23]). All of these forces are at work around the world, with developed countries such as the United States, Germany, Japan and France much further along in this change or transition than developing countries, such as India, Ghana and Brazil. With the increasing incidence of obesity and chronic diseases, the negative consequences of these changes, that is the shift from traditional to modern eating, has become more salient in the scholarly literature [3, 6, 7]. Diets have become homogenized and words like ‘Coca-Colonization’ have been used to describe the changes [7], see also [24]. In addition, advantages of traditional eating have been highlighted. For instance, it has been argued that traditional regional food consumption is a step towards sustainable rural development [25]. In addition, Trichopoulou [25] stated that traditional foods are environmentally friendly because they are often plant-based and integrated in the local biosystem, although there are certainly also animal-source traditional foods [26].

The change from traditional to modern eating has also been seen as a net negative by many in the general public and the media. In his New York Times bestseller “Food Rules” [27], Michael Pollan states “Regard nontraditional foods with skepticism” as one rule for eating wisely (p. 91). According to Pollan [27], “people who eat according to the rules of a traditional food culture are generally healthier than those of us eating a modern Western diet of processed foods” (p. 89). There are some signs of a return to traditional eating. Specifically, there seems to be a growing interest in sustainable food consumption, with some commonalities to traditional eating: Low meat consumption, low food waste, and high consumption^1^ of local foods were both labeled as sustainable (see Sustainable Development Goals [28]) and traditional [3, 6, 8, 29]. This growing interest is underlined by the terms sustainability, climate change, and environmental friendliness having joined the public discourse. Also, the interest in sustainable food has become a new source of income for the food industry. For instance, foods labeled as sustainable or local are common in Western supermarkets today and there are headlines such as “Europe’s food sector shows highest growth of sustainable product sales” [30]. Whether one considers the massive changes in eating behavior a net positive or negative, there is no doubt that a shift from traditional to modern foods and eating has occurred and that this is a timely and increasingly important topic.

However, what exactly is traditional and modern eating? Importantly, whereas changes in eating behavior are measurable, such as the intake of nutrients across time, what is considered traditional and modern eating mostly appears to be subject to a consensus agreement. Specifically, how much increase in a specific eating behavior over time is necessary to define this eating behavior as modern? What absolute level of a specific eating behavior then and now is necessary to call it traditional or modern? Hence, we believe that it is subject to human evaluation whether something is considered traditional or modern, and that this holds for both experts and lay people.

Moreover, what is considered traditional and modern eating varies across time, society, and culture. For instance, what is called modern in 2018 might be called traditional in 2100. Similarly, a food (e.g. sushi) might be perceived traditional in one country (e.g. Japan), but modern in another country (e.g., Germany). The latter example shows that, within a certain time, society, and culture, one might even talk about three categories when taking the perspective of foods: historically traditional, imported traditional, and modern. For instance, sushi might be considered ‘historically traditional’ in Japan, ‘imported traditional’ in Germany, whereas a new type of breakfast cereal might be considered ‘modern’ in both countries. However, the present article takes the perspective of people in a society or culture, for whom the consumption of ‘imported traditional’ foods might be nevertheless a ‘modern’ behavior, rendering two categories, namely ‘traditional’ and ‘modern’ eating behavior.

As far as it concerns these two categories, taking the perspective from 2018 and compiling international views, the literature indicates that multiple definitions of traditional and modern eating exist, rendering it complex and multifaceted. For instance, an often-applied definition of traditional and modern eating focuses on what people eat. Specifically, in scientific articles, modern diets have been defined by a high consumption of meat, sugar, oils, and fats [1, 3, 5, 6, 8–10, 31]. In contrast, traditional diets have been defined by a high intake of fiber and grains [3, 6, 8–10]. However, comparing today’s eating in many Western societies to how it was 100 years ago, one finds that there are not only differences in what people eat but also in how they eat, for example, whether people eat at home or in other places [3, 4]. This ‘how’-dimension of traditional eating has received considerably less research attention. Furthermore, a comprehensive compilation and systematization of these different facets has not yet been conducted and, thus, research in this area is impeded. This article aims to fill in this gap by comprehensively compiling and systematizing the different facets that are suggested to underlie traditional and modern eating. Moreover, we aim to present a comprehensive framework of traditional and modern eating across societies and cultures.

## Method: conceptualizations of traditional and modern eating

A qualitative approach was chosen to meet the aims of the article. Specifically, facets were compiled from the previous literature and expert discussions. In an inclusive approach, everything that was mentioned to be part of traditional or modern eating was compiled as a facet. A single mention of a behavior as part of traditional or modern eating by one article or one expert was enough for it to be listed as a facet in the present work. The only specification was that the facets had to be broad enough to potentially apply to more than one country. Hence, single traditional dishes, like Schnitzel in Austria [26], were not included as facets.

First, we compiled facets of traditional and modern eating through an extensive literature review in 2017 and 2018. The literature review targeted articles that specified characteristics of traditional or modern eating. Something was extracted as a facet of traditional or modern eating if the article explicitly used words like ‘traditional’ or ‘modern’ in relation to the facet. Furthermore, if an article stated that there was a pronounced increase in the facet within the last century, this was extracted as a modern facet. For instance, Popkin & Gordon-Larsen [6] stated that “modern societies seem to be converging on a diet high in saturated fats, sugar, and refined foods …” (p. S2). Hence, we extracted the facets ‘high consumption of saturated fats, sugar, and refined foods’ to characterize modern eating. The facets were extracted from the articles and saved together with the referencing article. The literature review was performed by one reviewer (GS) in major databases (e.g., Web of Science, PsycINFO, Google Scholar). Several combinations of the terms traditional, modern, food, eating, and nutrition transition were used. Also, references of relevant articles were screened and scientific books were reviewed. No limits were established regarding the year of publication. However, only articles published in peer-reviewed academic journals or scientific books were included. Amongst these, any type of article or review was included. Hence, we did not limit the literature review to empirical findings showing that something is part of traditional or modern eating. Instead, when authors of a manuscript mentioned something as part of traditional or modern eating, that was sufficient to be included as a facet of traditional and modern eating. A further inclusion criterion was English, French, or German as the article’s language.

Second, to prevent bias due to most literature targeting Western countries [32], we included facets that resulted from discussions within our group, whose members combine expertise from ten different countries. Specifically, we included perspectives from the USA (PR, MR, NA), Mexico (MK), Brazil (MA), France (CF), Germany (GS, BR, HS), Ghana (CA), Turkey (GK), India (RB, UM), China (XH), and Japan (SI, IF). Criteria for approaching the members of our group were being an academic and native of one of these countries, and well informed about eating in their native countries. Besides that, some members of our group had already collaborated in other cross-cultural food-related projects in the past which prompted to approach them for the present study. Our international group with interdisciplinary research experience draws on expertise in the psychology, anthropology, and sociology of eating, as well as nutrition and epidemiology.

Criteria for the selection of countries were diversity in terms of cuisines, obesity prevalence, income, and geography. The cuisines of these countries are characterized by distinct flavor principles. Specifically, the Mexican flavor principle is marked by tomatoes, onions, and chili peppers; the Japanese by soy sauce, sugar, and rice wine vinegar; the German by sour cream, vinegar, dill, mustard, and black pepper; the French by butter, cream, wine, and boquet garni; the Chinese by soy sauce, rice wine, and ginger root; the Brazilian by chili peppers, dried shrimp, ginger root, and palm oil; the Indian by garam masala; the Ghanaian by tomatoes, onion, and chili peppers sautéed in palm oil; and the Turkish by hot and intense spices [33, 34]. In addition, the US American cuisine constitutes a unique mixture of different ethnic groups [35]. Moreover, obesity prevalence in these countries differs and is displayed in Fig. 2. Specifically, obesity prevalence ranged from 3.4% in India to 36% in the USA in 2014 [37]. Furthermore, six of the countries (India, Ghana, China, Brazil, Mexico, Turkey) are considered middle-income countries, whereas the remaining four countries are considered high-income countries (range in GDP/capita from $2016 in India to $62,641 in the USA [38]). In addition, the ten countries cover five different continents (North America, South America, Africa, Europe, and Asia) and different climates, namely the equatorial climate (Ghana, Brazil, Mexico, India), the arid climate (USA, Mexico, India, China), the warm temperature climate (Germany, France, USA, Mexico, Brazil, Turkey, India, China, Japan), the snow climate (USA, Turkey, China, Japan) and the polar climate (China [39]).

Discussions took place in formal meetings about what constitutes traditional and modern eating in the respective countries. Specifically, based on the literature review a first list of facets was put together and presented to nine of our group (below referred to as ‘experts’) in a first face-to-face meeting. GS facilitated this meeting asking the experts about any missing facet in this list. Based on the experts’ feedback, the first list was extended, resulting in a second list of facets. This list was subsequently sent to all experts via email for reviewing and adding any facet that was missing. If necessary, GS held an online face-to-face meeting with an expert to clarify specific points. The feedback from all experts was incorporated into the facets list, resulting in a third list. This third list was finally reviewed in a second face-to-face meeting with all experts resulting in a fourth and final list of facets. This final list includes a compilation of 106 facets of traditional and modern eating (see Table 1).

**Table 1:** Facets of traditional and modern eating mentioned in previous research and in our group discussions as well as their assignment to the 12 subdimensions and 2 dimensions

Facets	Source (Reference; D = Group discussion)	T/M^a^
Dimension What People Eat		
Subdimension Ingredients		
High consumption of energy-dense foods	Dubé et al. (2014) [31]; Monteiro et al. (2013) [1] D	M
Consuming diet drinks or foods	D	M
High consumption of refined foods	Chopra et al. (2002) [10]; Popkin (2003) [8]; Popkin & Gordon-Larsen (2004) [6]; Popkin et al. (2012) [3]	M
High consumption of basic foods like wheat, corn, or rice	D	T
High consumption of animal-source foods	Popkin (2003) [8]; Popkin & Gordon-Larsen (2004) [6]; Popkin et al. (2012) [3]	M
High consumption of plant-based foods	D	T
High consumption of grain	Chopra et al. (2002) [10]; Drewnowski & Popkin (1997) [5]	T
High consumption of fruit	Dubé et al. (2014) [31]	T
High consumption of vegetables	Dubé et al. (2014) [31]; Popkin et al. (2012) [3]	T
High consumption of fiber	Chopra et al. (2002) [10]; Dubé et al. (2014) [31]; Popkin (2003) [8]; Popkin & Gordon-Larsen (2004) [6]	T
High consumption of sugar and caloric sweeteners	Chopra et al. (2002) [10]; Drewnowski & Popkin (1997) [5]; Dubé et al. (2014) [31]; Monteiro et al. (2013) [1]; Popkin (2003) [8]; Popkin (2009) [9]; Popkin & Gordon-Larsen (2004) [6]; Popkin et al. (2012) [3]	M
Consuming artificial sweeteners (e.g., in diet drinks, to sweeten coffee or tea)	D	M
High consumption of oils and fats (especially trans fats and saturated fats)	Chopra et al. (2002) [10]; Drewnowski & Popkin (1997) [5]; Dubé et al. (2014) [31]; Monteiro et al. (2013) [1]; Popkin (2003) [8]; Popkin (2009) [9]; Popkin & Gordon-Larsen (2004) [6]; Popkin et al. (2012) [3] D	M
High consumption of salt	Monteiro et al. (2013) [1]; Popkin et al. (2012) [3]	M
Subdimension Processing		
High consumption of industrially unprocessed foods	Monteiro et al. (2011) [40]; Popkin (2009) [9]	T
High consumption of fresh foods	D	T
High consumption of industrially ultra-processed foods	Monteiro et al. (2013) [1]; Popkin (2003) [8]; Popkin (2009) [9] D	M
Eating foods that are industrially mass-produced	Trichopoulou et al. (2007) [29]	M
High consumption of convenience products	Jabs & Devine (2006) [41]	M
Consumption of ultra-processed microwavable or frozen meals that were industrially produced	D	M
Consumption of fast foods	Jabs & Devine (2006) [41]	M
Consumption of soft drinks	Dubé et al. (2014) [31]	M
Eating foods with organic label	D	M
Subdimension Preparation		
High consumption of foods that require a long preparation/cooking time	D	T
Knowing how to cook	D	T
High consumption of foods that was cooked by a woman	D	T
High consumption of foods that has been prepared at home	Jabs & Devine (2006) [41] D	T
Eating home-canned foods	D	T
Eating foods that have been prepared in grandmother’s way	Vanhonacker et al. (2010) [42]	T
Flavoring most of the foods in a way that is typical for your country/region	D	T
Consumption of foods that are seasoned at the table (e.g., with salt, pepper)	D	T
High consumption of foods that were prepared using time-saving preparation equipment such as microwave ovens, rice cookers, and bread machines	Jabs & Devine (2006) [41]	M
Availability of a lot of different ways to cook/heat up foods	D	M
High consumption of fried foods	Popkin (2009) [9]	M
High consumption of grilled foods	Popkin (2009) [9]	M
High consumption of ready-prepared foods	Jabs & Devine (2006) [41]	M
Eating take-away or delivered meals	Popkin (2009) [9] D	M
Subdimension Temporal Origin		
High consumption of foods that have been eaten since the second World War	Trichopoulou et al. (2007) [29]	T
High consumption of foods that were known already by grandparents	D	T
High consumption of typical dishes	D	T
High consumption of foods from other countries’ cuisines	D	M
Eating pizza	Pingali (2006) [43] D	M
High consumption of foods that are recently produced	D	M
Consuming genetically modified foods	Lusk et al. (2005) [44]	M
Subdimension Spatial Origin		
High consumption of local food products	Trichopoulou et al. (2007) [29] D	T
High consumption of seasonal foods	D	T
Consumption of global food products from mass production	Trichopoulou et al. (2007) [29]; Popkin et al. (2012) [3]	M
Food available everywhere	D	M
Buying most foods at markets or small family stores	D	T
High consumption of cheap food products from supermarkets; especially cheap meat products	D	M
All foodstuffs are purchased (as opposed to grown or raised by oneself)	D	M
Eating foods from vending machines	D	M
Subdimension Variety		
Eating a diverse and varied diet	Drewnowski & Popkin (1997) [5]	M
Large number of food choices	D	M
Eating a large variety of different flavors	D	M
Eating a large variety of different types of fruits and vegetables	D	M
Eating a large variety within one type of fruit or vegetable	D	T
Dimension How People Eat		
Subdimension Temporal Aspects		
Taking time for eating	D	T
Eating an entire meal within 10 min or less	D	M
Regular/fixed mealtimes	Fjellström (2004) [45]	T
Eating at the same time in a family	D	T
Eating at traditional mealtimes	Mestdag (2005) [46] D	T
Consumption of main meals	Fjellström (2004) [45] D	T
Snacking	Mestdag (2005) [46]; Popkin (2009) [9]; Zizza et al. (2001) [47] D	M
Irregular/flexible mealtimes; skipping meals	D	M
Consumption of traditional dishes at celebrations/special occasions (e.g., Sundays, festivals)	D	T
Subdimension Spatial Aspects		
Eating at home	Jabs & Devine (2006) [41]; Popkin (2003) [8]; Popkin et al. (2012) [3] D	T
Eating out of home	Popkin (2009) [9]	M
Eating in restaurants	Jabs & Devine (2006) [41]; Story et al. (2008) [4]	M
Eating in buffet restaurants	D	M
Eating on the run	Jabs & Devine (2006) [41]; Mestdag (2005) [46]	M
High consumption of foods to go	D	M
Eating while working	D	M
Subdimension Social Aspects		
Eating together/ in company	D	T
Eating with family	Jabs & Devine (2006) [41]; Mestdag (2005) [46] D	T
Eating with colleagues	D	M
Eating alone	Fischler (2011) [48]; Kwon et al. (2018) [49]	M
Highly constraining, homogeneous collective rules	Fischler (1990) [50]	T
Eating is guided by social norms (Heteronomy)	Fischler (1990) [50]	T
Eating the same foods as the others when eating at home	D	T
Individualistic	D	M
Men get preferential treatment over women at mealtimes	D	T
Eating while being served foods by others	D	T
Larger family events center on meals	D	T
Having conversations while eating	D	T
Subdimension Meals		
Lunch or dinner as main meal of the day	D	T
Meals end with a sweet dessert	D	T
Foods that are eaten for breakfast differ largely from foods that are eaten for other meals	D	M
Drinking soft drinks during the main meal (e.g., cola)	D	M
Consumption of larger portion sizes	Benson (2009) [51]	M
Subdimension Appreciation		
Appreciation of foods	D	T
More food waste	D	M
Dissociation: not knowing where foods come from, and what is in them	D	M
Table manners	D	T
Eating in a way that shows respect for others at the table	D	T
Doing something else while eating	Jabs & Devine (2006) [41]	M
Using plastic utensils (e.g., plastic forks)	D	M
Subdimension Concerns		
Major concern: availability and quantity of food	Fischler (1990) [50]	T
Concern about whether foods are spoiled	D	T
Major concern: quality of food	Fischler (1990) [50]	M
Intuitive eating	D	T
Analytical eating	D	M
Interest in nutrition and consumer education	D	M
Interest in food & health labels	D	M
Trouble deciding what to eat	Fischler (1990) [50]	M
Concerns about eating too much	D	M

Note. ^a^ T refers to when a facet was mentioned as part of traditional eating by the respective reference(s) or in the group discussions; M refers to when a facet was mentioned as part of modern eating respectively

**Fig. 1:**
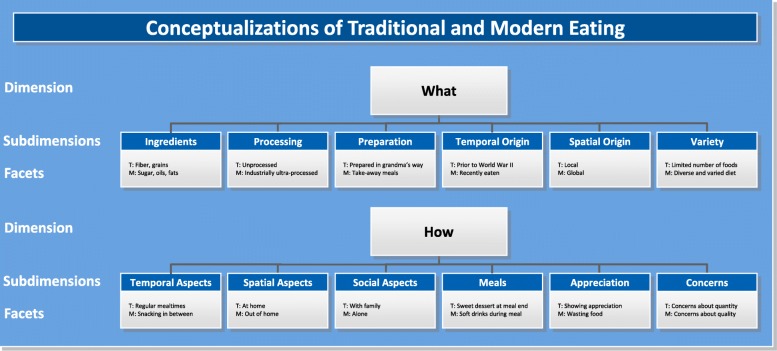
The TEP10 framework of traditional and modern eating, displaying dimensions, subdimensions, and examples of facets of traditional (‘T’) and modern (‘M’) eating

Third, an iterative process based on the constant comparative method of qualitative data analysis was used to implement a grounded theoretical approach [52]. Steps in the analytic process were (1) to classify a first set of the 106 facets into emergent categories, (2) to compare the remaining facets with these categories, and (3) to classify these facets into the existing categories and, if necessary, to revise these categories or to generate new ones. This process resulted in the classification of the 106 facets into 12 subdimensions, six of which were further subsumed under the dimension ‘what people eat’, and six of which were subsumed under the dimension ‘how people eat’ (see Fig. 1). As this research was part of a larger project, the Traditional Eating Project: 10 countries (TEP10; funded by the German Research Foundation, Grant SP 1610/2–1, granted to GS), the framework is called TEP10 framework.

## Results

### Dimension ‘what people eat’

The first dimension represents what people eat and includes six subdimensions, namely Ingredients, Processing, Preparation, Temporal Origin, Spatial Origin, and Variety.

#### Ingredients (subdimension 1)

A major aspect that differentiates traditional and modern eating is food ingredients. Fourteen facets were subsumed in this subdimension. For instance, the literature review and authors’ discussions revealed that traditional diets are characterized by a high consumption of basic foods,^2^ plant-based foods, grains [5, 10], fruit [31], vegetables [3, 31], and fiber [6, 8, 10, 31]. In contrast, modern diets are characterized by a high consumption of both energy-dense foods [1, 31] and diet drinks and foods. Moreover, modern eating includes a high consumption of refined foods [3, 6, 8, 10], animal-source foods [3, 6, 8], sugar and caloric sweeteners [1, 3, 5, 6, 8–10, 31], artificial sweeteners, oils and fats (especially trans fats and saturated fats [1, 3, 5, 6, 8–10, 31]), and salt [1, 3].

#### Processing (subdimension 2)

A second subdimension is the manner of production as well as the level of processing of foods. Nine facets were subsumed in this subdimension. Specifically, traditional diets are characterized by a high consumption of industrially unprocessed [9, 40] and fresh foods whereas modern diets are characterized by a high consumption of industrially mass produced [29] and ultra-processed [1, 8, 9] foods. In their NOVA classification, Monteiro et al. [54] categorize foods into the four groups ‘Unprocessed or minimally processed foods’, ‘Processed culinary ingredients’, ‘Processed foods’, and ‘Ultra-processed foods’. Ultra-processed foods “are not modified foods but formulations made mostly or entirely from substances derived from foods and additives” (p. 9 [54]). Examples of ultra-processed foods are subsumed in this subdimension, such as a high consumption of convenience products [41], ultra-processed microwavable or frozen meals that were industrially produced, fast food [41], and soft drinks [31] (please see [55] for an example how foods are categorized in the four groups). Foods that are labeled as organic were also discussed as part of modern eating with the emphasis on the label being modern, not necessarily the way of production.

#### Preparation (subdimension 3)

This subdimension refers to both who prepares the food as well as where and how the food is prepared. Fourteen facets were subsumed in this subdimension. For instance, consumption of home-made food [41] that was prepared by women is considered part of traditional eating. Regarding how the food is prepared, traditional foods require a long preparation time as well as are prepared as one’s grandmother would have done [42]. In contrast, modern eating is defined by the use of time-saving food preparation equipment such as microwave ovens, rice cookers, and bread machines [41], and by a lot of different ways to cook and heat up foods (e.g., frying, boiling, steaming, grilling). Also, high consumption of fried and grilled foods can be considered modern [9] as well as a high consumption of ready-prepared food [41] or take-away/delivered meals [9].

#### Temporal origin (subdimension 4)

The fourth subdimension that we identified includes facets that refer to the length of time that a food has been part of the diet in any particular region. Seven facets were subsumed in this subdimension. For instance, foods that are typical for the region or foods present for a long time (e.g., before the Second World War, as suggested by Trichopoulou and colleagues [29]) are considered as traditional. Our discussions revealed that a high consumption of foods that were already known by people’s grandparents is another facet in this subdimension. Weichselbaum, Benelam, and Soares Costa [26] published a synthesis report listing such traditional foods across Europe. For instance, Wiener Schnitzel is considered a traditional food in Austria, Pumpernickel bread in Germany, Cured Greenland shark in Iceland, and Kebab with yogurt in Turkey [26].

#### Spatial origin (subdimension 5)

This subdimension has to do with where the consumed foods come from. Eight facets were subsumed in this subdimension. For instance, traditional eating is defined as a seasonally restricted and local food consumption [29]. In contrast, modern eating is characterized by consumption of foods that are imported from all over the world [3, 29], and are therefore available for consumption throughout the year. Moreover, authors’ discussions revealed that, traditionally, foods were primarily bought at farmers’ markets or grown by oneself whereas in modern times, foods are mostly bought in supermarkets, in convenience stores, or from vending machines.

#### Variety (subdimension 6)

Within this subdimension, modern eating is characterized by a large choice of available foods. Five facets were subsumed in this subdimension. One example facet is a diverse and varied diet [5]. This variety may be especially pronounced regarding the availability of different flavors. Also, eating a variety of different types of fruits and vegetables was discussed to be part of modern eating (e.g., apples, bananas, grapes), being able to eat them year-round via imports from countries with different climate. Notwithstanding, diversity within one type of fruit or vegetable may be part of traditional eating (e.g., eating different kinds of local apples).

### Dimension ‘how people eat’

The second dimension represents how people eat and includes the six subdimensions: Temporal Aspects, Spatial Aspects, Social Aspects, Meals, Appreciation, and Concerns.

#### Temporal aspects (subdimension 1)

The first subdimension that we identified includes duration of eating and when people eat. Nine facets were subsumed in this subdimension. Specifically, it was discussed that, traditionally, people take time^3^ to eat. In addition, Fjellström [45] and Mestdag [46] stated that, traditionally, people eat main meals at regular and traditional mealtimes. Moreover, our group’s discussions revealed that, in many countries, it is traditional for all family members to eat together at the same time. Also, traditional dishes are often consumed on special occasions (e.g., Sundays, festivities). In contrast, modern eating has been discussed to be characterized by a shorter eating duration, by eating irregularly, and by skipping meals. Moreover, Zizza et al. [47] consider snacking between meals as part of modern eating.

#### Spatial aspects (subdimension 2)

This subdimension focusses on where people eat. Seven facets were subsumed in this subdimension. For instance, traditional eating is characterized by eating at home [3, 8, 41]. In contrast, eating in restaurants is modern [4, 41], especially in buffet restaurants. Moreover, eating on the run is categorized as part of modern eating in the USA [41]. Also, eating food ‘to-go’ (i.e., take-away food) as well as eating while working was classified as modern.

#### Social aspects (subdimension 3)

A third subdimension is with whom people eat, and the extent to which social norms are present and followed. Twelve facets were subsumed in this subdimension. Specifically, eating together, especially with the family, is part of traditional eating [41, 46]. Also, meals are traditionally central opportunities for conversations in many countries and are at the center of larger family events. In contrast, in modern times, people more often eat by themselves [48]. As another social aspect, Fischler [50] mentions that traditionally, eating is guided by social norms and highly constraining, homogeneous collective rules. As a result, everybody eats the same food within a meal at home. One of these rules, which is present in many countries, is that, traditionally, men get preferential treatment over women at mealtimes. For instance, men eat while women serve food in India, Ghana, and Mexico. In comparison, modern eating is more individualistic and egalitarian, and based on individual preferences rather than on social norms [50].

#### Meals (subdimension 4)

Another subdimension that we identified was the significance and content of meals, such that some meals consistently feature particular content, and some meals during the day are considered more important and substantial than others. Five facets were subsumed in this subdimension. For instance, which meal is considered the main meal of the day is a discriminant feature between traditional and modern eating. For example, traditionally, the main meal is lunch in Germany, whereas in modern times the main meal is dinner.^4^ Regarding the content of meals, traditionally, Western main meals end with a sweet dessert. In contrast, drinking soft drinks during the main meal was considered to be modern, as well as consuming special foods for breakfast that differ largely from the foods eaten at other meals.

#### Appreciation (subdimension 5)

This subdimension targets the extent to which respect is shown for the food consumed, as well as for other people at the table. Seven facets were subsumed in this subdimension. Specifically, authors’ discussions revealed that traditional eating is characterized by the appreciation of food and adhering to table manners, that is to eat according to socially accepted conventions. In contrast, modern eating is marked by wasting food (e.g., throwing away the rest of a meal instead of eating it later), using plastic utensils, and not knowing where the food comes from or what is in it. Also, doing something else while eating is part of modern eating (e.g., watching screens [41]).

#### Concerns (subdimension 6)

The sixth subdimension deals with concerns about eating. Nine facets were subsumed in this subdimension. For instance, traditional eating is characterized by concerns about the availability of food, whereas, in modern times, concerns center on the quality of food [50]. Also, traditionally, people eat in an intuitive way, whereas modern eating is often marked by an analytical approach. Specifically, people pay attention to nutritional aspects and food labels. Scrinis [56] has labeled this focus on nutrients as ‘nutritionism’. In the light of the variety and abundance of the modern food environment, people are concerned both about what to eat [50] and about eating too much.

## Discussion

The TEP10 framework summarizes a comprehensive compilation and systematization of the different facets that are suggested to underlie traditional and modern eating. It shows that traditional and modern eating is characterized not only by what people eat, but also by how they eat. Twelve subdimensions and 106 facets were suggested to underlie traditional and modern eating. Therefore, the current study provides a broad overview of what constitutes the concept of traditional and modern eating.

Importantly, the present framework shows that traditional and modern eating is complex and multifaceted. It is not only defined by one facet, such as eating traditional dishes, but by the co-occurrence of multiple facets at the same time, such as eating traditional dishes on Sundays together with the family. This co-occurrence might be the critical factor in finding evidence for the relationship between traditional and modern eating and health. Specifically, certain facets might need to come together to have an effect on health outcomes. For instance, foods with traditional temporal origin, such as Wiener Schnitzel in Austria [26], might need to be eaten according to traditional temporal aspects, such as only at special occasions. Also, it is possible that a combination of some modern and some traditional facets has health effects. For instance, eating a wide variety of different types of fruits and vegetables (modern) as part of a family dinner at home (traditional) might have a health effect. The presented framework enables both the differentiated examination as well as the investigation of the joint impact and interplay of different facets on health outcomes.

**Fig. 2:**
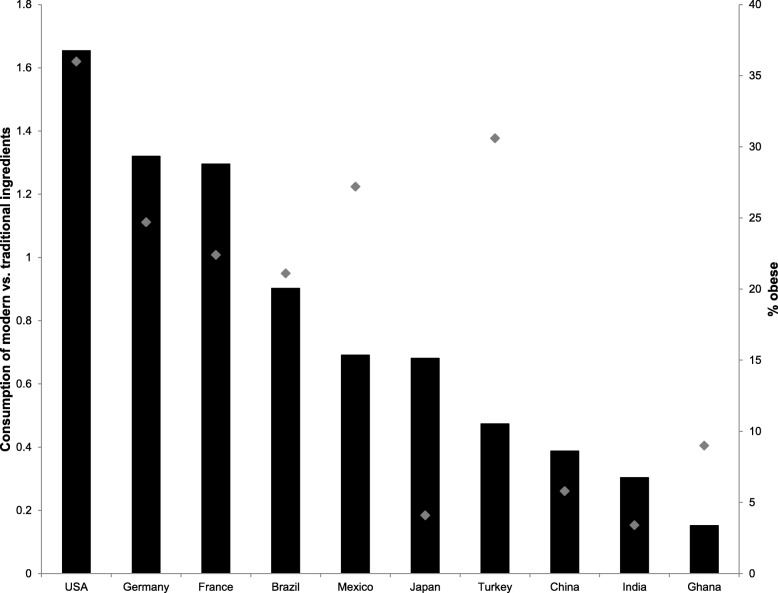
Bars represent the quotient of percentage of energy derived through ‘modern vs. traditional ingredients’ with data from the FAO [36]. Points depict the prevalence of obesity in 2014 (i.e. BMI ≥ 30 kg/m^2^) [37]. Note. Cereals, starchy roots, pulses, vegetables and fruits were considered to be ‘traditional ingredients’ whereas sugar/sweeteners, meat/offal, and vegetable oils/animal fats were considered to be ‘modern ingredients’

The potential of a joint examination of multiple facets of traditional and modern is displayed in Fig. 2. Specifically, for ten selected countries, the co-occurrence of ‘modern vs. traditional ingredient’^5^ consumption and obesity prevalence is displayed in Fig. 2. The ‘modern vs. traditional ingredient consumption’ that is displayed on the left Y-Axis of Fig. 2 is calculated with data from the Food and Agriculture Organization of the United Nations [36]. Specifically, we computed the percentage of consumed energy that comes from ‘modern ingredients’ divided by the percentage of energy that comes from ‘traditional ingredients’. As a high consumption of cereals, vegetables, and fruits was reported to be part of traditional eating [3, 10, 31], these were regarded as ‘traditional ingredients’. Similarly, a high consumption of sugar/sweeteners, meat/offal, and vegetable oils/animal fats was reported to be part of modern eating [1, 6, 8, 9]; therefore these were regarded as ‘modern ingredients’. With values higher than 1, people in the USA, Germany, and France derive more energy from ‘modern’ than from ‘traditional’ ingredients, whereas the opposite is true for Brazil, Mexico, Japan, Turkey, China, India, and Ghana with values below 1. As can be seen, across these ten countries, the co-occurrence of modern vs. traditional ingredients consumption is related to obesity prevalence (*r* = .68). It is, however, important to note that such a relationship with obesity prevalence might be absent or even reversed for other subdimensions or facets of traditional and modern eating.

As for the relationship between traditional eating and health outcomes, the TEP10 framework shows that there are two further issues that need to be considered. First, this relationship needs to be investigated in relation to society, culture, and time. An example why this is important lies in ‘imported traditional’ foods which were considered to be part of modern eating in the adopting society or culture. However, these imported foods probably have similar nutritional qualities to those from traditional cuisines. Hence, given that the consumption of sushi can be considered traditional in Japan but modern in Germany, the ingested nutrients of a German ‘modern eater’ who eats a lot of sushi are comparable to a Japanese ‘traditional eater’ who does so. This demonstrates that general statements about the relationship between traditional eating and health are rarely tenable but need to be related to society, culture, and time.

Second, the TEP10 framework shows that a simple dichotomy between traditional and modern eating is an oversimplification, even within a certain time, society, or culture. Specifically, a person might score high on traditional eating regarding one facet or subdimension but high on modern eating regarding another facet or subdimension. For instance, an Italian who consumes a lot of frozen mass-produced pizza would score high on traditional eating with regard to the Temporal Origin subdimension, as pizza has been labeled traditional in Italy [57]. However, he or she would score high on modern eating with regard to the Processing subdimension as mass-production has been classified as modern [29]. This shows again that generic statements about the relationship between traditional eating and health outcomes are difficult to support. Rather, statements about the relationship between certain facets of traditional eating or their co-occurrence and health are possible.

The multidimensionality of traditional and modern eating also underlines its conceptual distinction from sustainable and healthy eating. Specifically, although low meat consumption, low food waste, and high consumption of local foods seems to be part of both sustainable (see Sustainable Development Goals [28]) and traditional eating [3, 6, 8, 29], traditional eating was defined by many other facets. In a similar vein, a high intake of fruits, vegetables, unprocessed and fresh foods as well as a low intake of fat, sugar, and salt seems to be both part of traditional [1, 3, 5, 6, 8–10, 31, 40] and healthy eating [58]. However, traditional eating goes beyond the consumption of these foods and also includes how people eat.

As far as it concerns healthy eating, the TEP10 framework shows a new perspective on modern eating. Specifically, a frequently mentioned characteristic of modern eating is that there is a focus on nutrients (‘nutritionism’, [56]) and concerns about the healthiness of foods coexist with a high consumption of ‘modern’ ingredients that are considered to be unhealthy, such as sugar. Specifically, Rozin et al. [59] showed that US-Americans scored highest on concerns about the healthiness of foods as compared to Belgians, French, and Japanese. At the same time, US-Americans also score highest on the intake of ‘modern’ ingredients such as meat, sugar, oils, and fats, as compared to the other three countries [36]. This paradox appears to be a central characteristic of modern eating. Therefore, we included concerns in the framework of traditional and modern eating, although one could argue that concerns do not qualify as ‘eating’.

The TEP10 framework allows a comprehensive and in depth investigation of traditional and modern eating in future research. Next to the investigation of consequences (e.g., for health), it also enables examination of the drivers of the transition from traditional towards modern eating. For instance, motives for why people eat what they eat [60–62] or what meaning food has for individuals [63] might be factors underlying the different facets of traditional and modern eating. The TEP10 framework offers both to comprehensively investigate traditional and modern eating as well as to focus on single facets, while acknowledging the multidimensionality of the overall phenomenon. Furthermore, the TEP10 framework enables researchers to uncover similarities and differences in the concept of traditional and modern eating across the world. In the case of Japan, we have already investigated whether the presented multidimensionality of traditional and modern eating is valid [64]. Specifically, we asked 340 adults from Japan to rate the ‘traditionality’ of 46 facets. The results showed that, in accordance with the TEP10 framework, traditional and modern eating is also multidimensional in Japan. More precisely, both dimensions what and how people eat are part of traditional and modern eating in Japan as well as ten subdimensions of the TEP10 framework [64].

There are some limitations and avenues for future research that need to be addressed. The presented compilation of facets constitutes a first step and is certainly a developing process with additional facets to be potentially included in the future, for example from countries that were not represented in this manuscript. Also, future research needs to add quantitative evidence whether the facets are part of traditional and modern eating; for instance, by surveying people about the ‘traditionality’ or ‘modernity’ of facets.

## Conclusion

The TEP10 framework is a step towards a comprehensive understanding of the concept of traditional and modern eating. Specifically, traditional and modern eating is not only characterized by what people eat but also by how they eat, a dimension that has been neglected in past research. The present article sheds new light on the overall phenomenon of traditional and modern eating, underlining its multidimensionality. Also, it shows that reducing traditional and modern eating to single dimensions, subdimensions, or facets constitutes an oversimplification of the overall phenomenon. Future research might benefit from considering the multidimensionality and interplay of multiple facets of traditional and modern eating. This might provide new insights into the transition from traditional towards modern eating, its consequences and underlying factors, moving forward research on this timely and important topic.

## Data Availability

Not applicable.

## Abbreviations

BMI: Body Mass Index
D: Group discussion
FAO: Food and Agriculture Organization of the United Nations
M: Modern
T: Traditional
TEP10: Traditional Eating Project: 10 countries

## Footnotes

1 Please note that with the term “high consumption” we refer to the overall intake across multiple eating occasions. Most often, this might mean a frequent consumption of the respective food but might also mean a high consumed amount in a single eating occasion in some cases.

2 Please note that the term ‘basic foods’ relates to a definition provided by The Department of Health of the Australian Government [53]: Basic foods provide the nutrients essential for life and growth. These foods are also known as ‘everyday foods’.

3 Please note that ‘taking time to eat’ and other terms within this manuscript are subjective and subject to interpretation.

4 Please note that this largely varies by country. For instance, in the USA the main meal is traditionally dinner.

5 Please note that the expression ‘modern vs. traditional ingredients’ is used for simplification. However, while some ingredients and foods are objectively modern (they did not exist in the past), what is specifically modern in many cases is not the food itself but how much and how often it is consumed.
